# Facilitating higher-order thinking with the flipped classroom model: a student teacher’s experience in a Hong Kong secondary school

**DOI:** 10.1186/s41039-017-0048-6

**Published:** 2017-02-10

**Authors:** Kin-yuen Lee, Yiu-chi Lai

**Affiliations:** The Education University of Hong Kong, Tai Po, Hong Kong

**Keywords:** Flipped classroom, Active learning, Higher-order thinking

## Abstract

This paper aims to discuss an exploratory study that a student teacher carried out on applying the “flipped classroom” approach in his information and communication technology (ICT) class. The study examined student perceptions of the new teaching approach and investigated whether it can help promote higher-order thinking. This study involved 28 students in a public secondary school in Hong Kong. They were attending an ICT class on 3D modelling. A mixed methods approach was adopted. Both quantitative and qualitative data were collected through surveys, online quizzes and focus group interview. The students’ assignments were also examined and analysed. The findings show that students are inclined to accept the new teaching model. We can conclude that it is possible to improve students’ higher-order thinking capability using the flipped classroom approach to teaching.

## Introduction

The Hong Kong Education Bureau (EDB) introduced its document on the first strategy on IT in education in 1998, which became the first step towards the widespread use of IT in Hong Kong education. Since then, many educators have expressed the belief that IT is a promising means to improve the quality of teaching and learning in every aspect. In 2014, the consultation document on the fourth strategy on IT in education, entitled “Realising IT Potential, Unleashing Learning Power: A Holistic Approach”, was released. It aimed to explore the possibility of developing the use of IT in education in the foreseeable future (Education Bureau 2014). It has been noted that cases of using the flipped classroom approach to enhance students’ achievement were mentioned in the document. Many advocates argue that this new teaching approach can help greatly improve the quality of teaching and learning (Bergmann and Sams 2012). Recently, many local teachers shared their experience of the “flipped classroom” in different workshops and seminars organized by relevant education organizations. This certainly inspires other teachers to adopt this new teaching approach in their classes. In fact, questions on “ways of flipping a classroom” and “student learning achievement in flipped classrooms” are still hot among educators at both local and global levels. However, not everyone holds a positive view of flipped classrooms: referring to the decline of Hong Kong’s Science PISA ranking in 2016, some educators argue that this new approach does not help improve students’ ability to engage in scientific inquiry. They also claim that e-learning practices in Hong Kong cannot help promote higher-order thinking (Ho and Lam 2016; Lo 2016). Their opinions are at odds with research findings on flipped classrooms (Lankford 2013; Nederveld and Berge 2015; Zainuddin and Halili 2016). In these circumstances, the present researchers attempted to conduct an empirical study to investigate student perception of flipped classrooms, and their link to higher-order thinking. This article will discuss the exploratory study done to illustrate how to implement the flipped classroom model and especially to examine whether it can nurture students’ higher-order thinking ability.

## The flipped classroom model and higher-order thinking

The term “flipped classroom” refers to a new teaching model which adopts a student-centred approach and enables teachers to rearrange class time and time for homework (Bergmann and Sams 2012; Johnson et al. 2014). In a typical flipped classroom, students will be asked to study online instructional materials such as videos, or do anything that helps them understand the subject content at home prior to classes. This arrangement allows teachers to allocate more class time to learning activities (Bergmann and Sams 2012). As the “flipped classroom” is greatly facilitated by making use of technology, it is often classified as an e-learning approach by educators. However, Bishop and Verleger (2013) criticized the term “flipped classroom”, arguing that is a buzz word and there is no underpinning rationale behind the new teaching model. They proposed that the flipped classroom must involve learning on computers independently, outside of the classroom, and interacting with classmates in small groups inside the classroom. Herreid and Schiller (2013) pointed out that the flipped classroom provides more flexibility for students’ learning, improves students’ learning achievements and allows creative and innovative teaching. Couch (2014) argued that students’ learning attitudes and academic achievements can be improved with the flipped classroom. Chua and Lateef (2014) suggested that the model is widely accepted by university students in Asia.

On the other hand, higher-order thinking is a perplexing concept and could be defined as the use of critical and creative thinking, which enables one to solve complex problems (Yeung 2012). Nurturing young students’ higher-order thinking capability is a major goal of the recent curriculum reform in Hong Kong and is crucial for a knowledge-based society (Leung 2013; Yeung 2012). Smith (2007) pointed out that asking students open-ended questions can engage them in making comparisons, providing justification or conducting inquiry based on prior knowledge. This helps develop their higher-order thinking skills. Apart from effective questioning strategies, Leung (2013) proposed that active learning strategies should be adopted to promote higher-order thinking. Research findings also show that higher-order thinking can be promoted through the flipped classroom as students can pause videos to think about the learning content (Hamdan et al. 2013; Herreid and Schiller 2013). Moreover, some researchers have suggested that Bloom’s taxonomy can be used as an effective protocol in assessing students’ knowledge (Hamdan et al. 2013; Khairuddin and Hashim 2008; See and Conry 2014; Yeung 2012). Lankford (2013) added that the flipped classroom model allows facilitators to use time primarily in the top layers of Bloom’s taxonomy like application, synthesis, evaluation and analysis. In addition, Nederveld and Berge (2015) argued that in flipped learning, instructors can spend classroom time on application and higher-level learning rather than on lecturing and other lower-level thinking tasks. They also pointed out that the approach gives instructors greater opportunity to detect errors in thinking, and supports creative problem-solving and effective communication. Zainuddin and Halili (2016) noted that classroom tools used in flipped learning, such as group discussion, allow students to spend more time on higher-level learning.

## Research questions and purposes

To acquire better understanding of the implementation of the flipped classroom model, and to examine how to use this approach to promote higher-order thinking, the following research questions were posed:

1. What are students’ perceptions of implementing the flipped classroom in public secondary schools in Hong Kong?
2. Is it feasible to promote higher-order thinking using the flipped classroom approach?

**Fig. 1:**
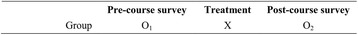
Design of the research

The study was conducted by a student teacher in a public secondary school in Hong Kong during his field experience block practice in the final year of his Bachelor’s degree. The school, located in a new town, mainly received students in the medium band—in Hong Kong, all primary six students are categorized in three equal bands by the EDB according to their academic performance when they are promoted to secondary schools. Twenty-eight participants were involved: secondary 2 students in the same information and communication technology (ICT) class. The students and their parents had agreed to join the study and allowed the student teacher to collect data for research purposes. Due to the limitations imposed by the field experience teaching arrangements, only one class participated in the study. Thus, the study adopted the “one group pretest-posttest” design (see Fig. 1) to examine the differences between “traditional classroom teaching” and “flipped classroom teaching” (Schreiber and Asner-Self 2010).

## Methods

The unit “3D Modelling: SketchUp” was being taught during the period of the study. Usually, this unit would be completed within four double lessons (35 minutes per lesson) over 4 weeks. “3D Modelling” is not a topic listed in the official junior form computer literacy curriculum (Curriculum Development Council CDC 1999). Since the current curriculum document has not been updated for nearly 16 years, many teachers criticize its contents as obsolete and prefer to design their own school-based ICT curriculum. Most think that a school-based curriculum can arouse students’ interest in learning ICT and unleash their creativity.

### Pre- and post-course surveys

The students were asked to complete the same survey before and after the course. The instrument was partly adapted from a similar one in Couch (2014). Couch’s survey focused on students’ experiences of science education and their understanding of their learning. In contrast, the surveys used in this study aimed to collect quantitative data in order to examine the following three aspects: (a) subject knowledge, (b) class participation, and (c) learning and teaching activities. The pre- and post-course surveys were validated by the school teacher and the student teacher’s supervisor prior to being given to the students, to ensure consistency with the required outcomes and provide face-validity.

### Design and sequence of the lessons

The flipped classroom version is refined from the lesson designed by an experienced ICT teacher in the same school. The original design adopted a traditional approach in which the class activities mainly comprised a 25-min demonstration session of the 3D modelling software and a 45-min hands-on practical session. However, when adopting the flipped classroom approach, the demonstration session was removed from the classroom time and replaced by online instructional videos. This arrangement gave the teacher more class time to organize student-centred learning activities. From Table 1, we can see that, in the first lesson, the teacher was able to arrange a discussion and a brainstorming session before and after the hands-on task. There was also sufficient class time for students to demonstrate their work in the second lesson and share their own design in the last lesson. Overall, the students had more time to complete their hands-on 3D modelling task in class. In addition, even when the teacher found that some students were unable to complete the pre-lesson video activity, they could watch the instructional videos within 10 min before starting the hands-on tasks. Table 1 shows the timeline of the learning activities and data collected from the students.

**Table 1:** Timeline of the learning activities and data collected from the students

Event	Unit topic	Learning activity/data collection
1 week before the start of a unit		• Pre-course survey
Lesson 1	Basic operation of SketchUp	Before the lesson • Watching online instructional videos • Online quiz During the lesson • Class discussion: Examples of 3D chair model were presented to help students understand the task • Assignment: Students were required to follow procedures described in the handout and create a simple 3D chair model for assignment 1 • Brainstorming: Students tried to formulate their own design with peers to create a new 3D model
Lesson 2	Other common tools and features of SketchUp	Before the lesson • Watching instructional videos • Online quiz During the lesson • Assignment: Students continued to work on assignment 1 and had to finalize the design • Student demonstration After the lesson • Assignment submission: Students were required to submit assignment 1 for grading before the start of lesson 3
Lesson 3	Advanced features of SketchUp	Before the lesson • Watching instructional videos • Online quiz During the lesson • Assignment: Students were required to design and create another 3D model (e.g. a house, cartoon character, car) for assignment 2
Lesson 4	SketchUp Project	Before the lesson • Watching instructional videos • Online quiz During lesson • Assignment: Students continued to work on Assignment 2 and had to finalize the design • Share own designs • Complete post-course survey After the lesson • Focus group interview

The student work was collected and analysed. This was an important qualitative data source that allowed the researcher to examine the students’ performance.

### Preparation of the instructional videos

Prior to this case study, the student teacher did not have in-depth knowledge of 3D modelling or experience of using SketchUp. It seemed that using tutorial videos already available on the Internet was a feasible solution. However, he found that those existing videos about 3D modelling techniques were not suitable for secondary 2 students. Moreover, there were no resources with traditional Chinese captions and/or Cantonese narration on the Internet. To solve this problem, he decided to prepare the instructional videos himself with the screen-casting application Adobe Captivate, based on the videos on a playlist entitled “New to SketchUp?” (https://www.youtube.com/playlist?list=PLF001616C0ADF4245) on the SketchUp YouTube channel. After gaining some experience of video production, the student teacher soon realized that a fully narrated video of more than ten minutes was too lengthy and discouraged students from watching.

### Designing and administrating online quiz

**Fig. 2:**
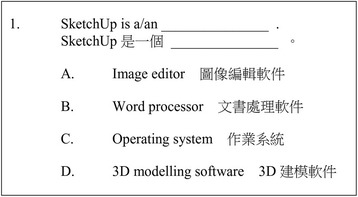
Assessment item for testing students’ basic knowledge (remembering)

**Fig. 3:**
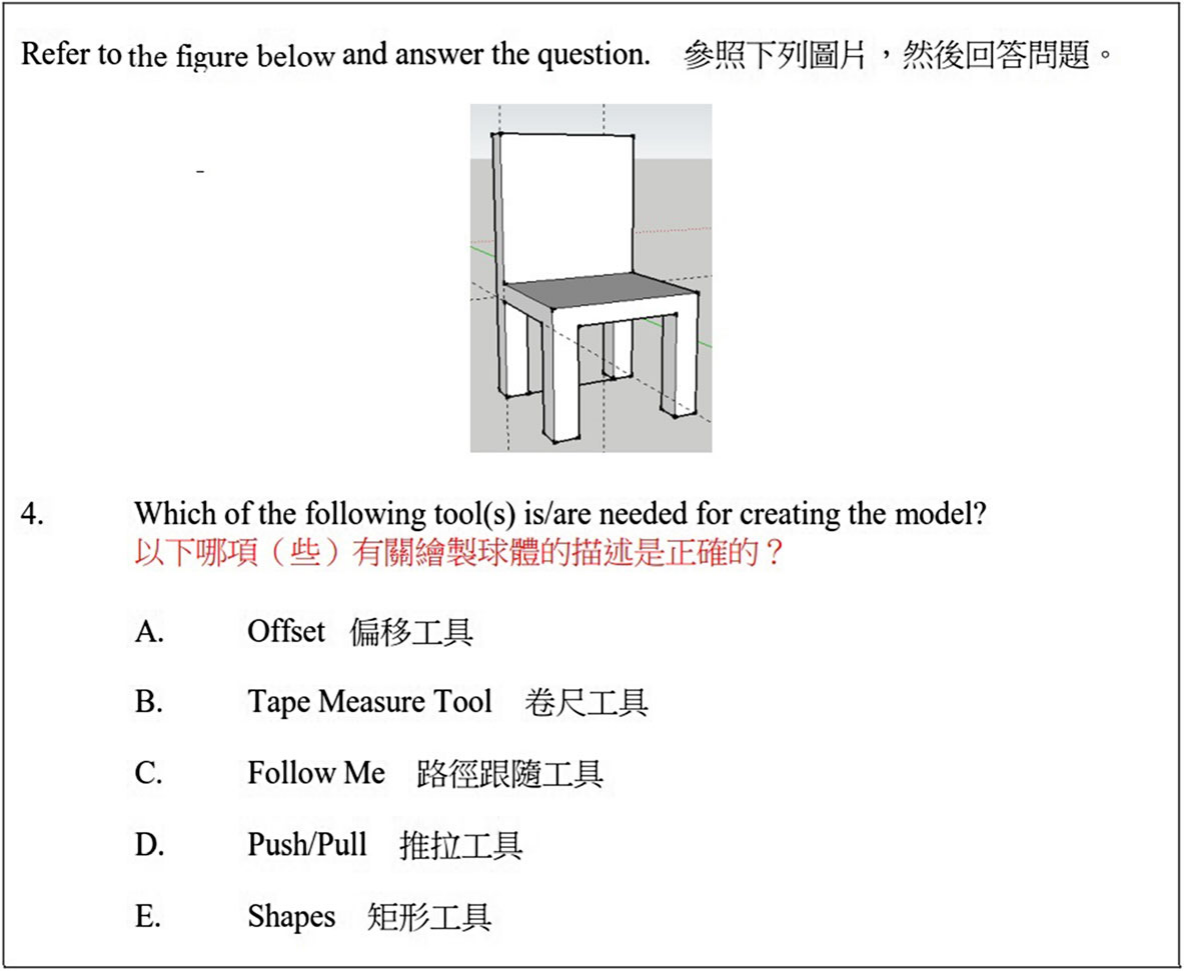
Assessment item for testing students’ understanding of skills in using 3D software (understanding)

**Fig. 4:**
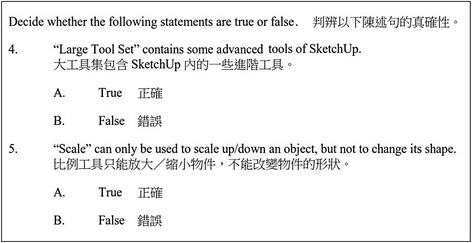
Assessment items for testing students’ higher-order thinking (analysing)

**Fig. 5:**
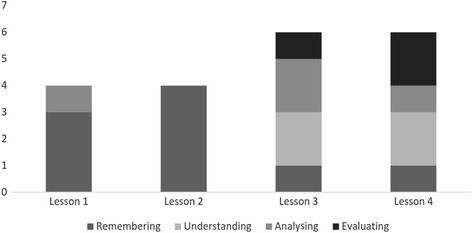
Number and types of questions in each online quiz

Each quiz consisted of a few questions about the subject content learnt in the lesson. Assessment items were set at different levels with reference to the revised version of Bloom’s taxonomy (Krathwohl 2002). Due to the limitation of the quiz design, only four categories of the taxonomy could be assessed: *remembering*, *understanding*, *analysing* and *evaluating*. The remaining two, *applying* and *creating*, were examined through other research instruments. The following figures show example questions in the quiz. Figure 2 shows a question in the *remembering* category, about the major function of SketchUp. The question in Fig. 3 is an example of the *understanding* category while that in Fig. 4 is an *analysing* example. Lai and Wong (2005) suggested that properly designed assessment items are useful for assessing and nurturing students’ higher-order thinking capability. Figure 5 presents the number of questions used in the quizzes in the four categories drawn from the revised Bloom’s taxonomy.

The online quizzes were validated by the student teacher’s supervisor prior to being given to the students to ensure consistency with the required outcomes and provide face-validity. The online quizzes were sources of qualitative data that helped analyse student performance.

### Delivering the learning materials

**Fig. 6:**
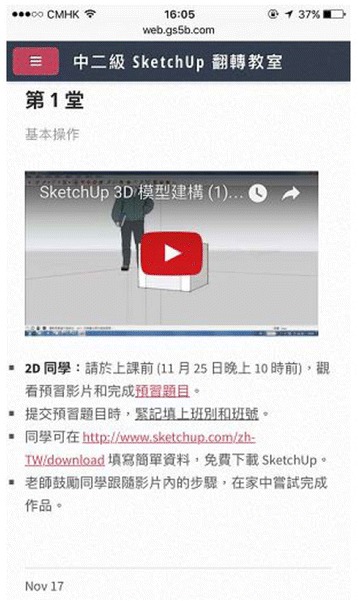
Video and reminders on the webpage for lesson 1

To make the instructional videos easily accessible, the student teacher uploaded them all to YouTube and created a webpage in which they were embedded. This webpage was intended to provide a one-stop learning platform. The platform adopted a responsive web design to make it more mobile-friendly. Videos were uploaded 2 to 3 days before each lesson so that the students would have sufficient time to prepare in advance (see Fig. 6).

Through the learning platform, the students could access all teaching materials, such as the teaching schedule, instructional videos and quizzes. Since the flipped classroom teaching approach was still new for the students, a printed copy of the reminder was distributed to students before the start of the first lesson. This reminder showed the link to the learning platform and helped remind the students to watch the videos at home before the lesson.

### Focus group interview

A semi-structured focus group interview was scheduled after the last lesson (see Table 1). It aimed to probe the students’ perception of the flipped classroom approach. The qualitative data helped to triangulate the data collected from other instruments. By collecting data through different instruments, it was possible to establish the trustworthiness of the data.

## Results and Discussion

### Pre- and post-course survey

#### Subject knowledge

Four survey items were used to investigate the students’ perception of their own subject knowledge. Table 2 shows the mean, standard deviation and *t* value of both pre-course and post-course scores. The data show that the mean scores of all items were higher after the course. However, only the change in item 3 (*analysing*/*evaluating*) is statistically significant. A possible reason for this is that all the students felt that they could start their own project based on their own ideas.

**Table 2:** Scores of items that assessed students’ perception of their own subject knowledge

Items	Number	Pre-course		Post-course		*t*	Sig.
		*M*	SD	*M*	SD		
1. In the lesson, I could follow the teacher’s instructions and finish learning tasks assigned by teacher. (Remembering/understanding)	26	3.58	.758	3.81	.801	−1.063	.298
2. I felt confident about applying the knowledge acquired in computer lessons in the future. (Applying)	26	3.46	.761	3.73	.724	−1.570	.129
3. In the lesson, I could use the knowledge and skills taught by the teacher to start my project and demonstrate creativity. (Analysing/evaluating)	26	3.58	.758	3.96	.662	−2.083	.048*
4. In the lesson, I could reflect on what I had learnt, attempt to complete the learning tasks or start my project with alternative method(s) not taught by the teacher. (Creating)	26	3.27	.533	3.65	.745	−1.995	.057

*p < .05

#### Class participation

Comparison of the pre- and post-course surveys shows that the mean scores of all items increased. Regarding student motivation, item 3 shows a change with statistical significance. This implies that most students were willing to engage in discussion with their classmates when their teacher used the flipped classroom approach. Students were also very positive about class support during lessons. This is reflected in the scores of items 4 to 6: the flipped classroom approach fosters a supportive learning environment. However, there is still no strong evidence to show that peer interaction was enhanced in this case study (see item 7 of Table 3).

**Table 3:** Scores of items assessing students’ perception of class participation

	Number	Pre-course		Post-course		*t*	Sig.
		*M*	SD	*M*	SD		
Motivation (*N* = 26)							
1. I attempted to engage in class activities.	26	4.12	.766	4.23	.710	−.721	.478
2. I was willing to ask my teacher questions.	25	3.72	.936	3.96	.790	−1.541	.136
3. I was willing to discuss with my classmates.	26	4.08	.744	4.35	.689	−2.273	.032*
Class support (during the lesson)							
4. I was provided with adequate time to complete the learning tasks.	28	3.29	.937	3.86	.891	−4.076	.000**
5. I could get help from my teachers.	28	3.86	.803	4.36	.559	−3.334	.002**
6. I had opportunities to discuss with my teachers.	27	3.85	.662	4.26	.594	−2.833	.009**
7. I had opportunities to discuss with my classmates	28	4.07	.663	4.21	.686	−1.279	.212

*p < .05, **p < .01

#### Usefulness of learning and teaching activities

Table 4 shows the results for students’ perception of the usefulness of learning and teaching activities. Before the course, the students had no preconceived ideas about the flipped classroom. Teachers normally demonstrate how to use software in class time. But, when adopting the flipped classroom model, the students watched instructional videos to learn how to use the software at home. Item 1 was used to examine their perception of their teacher’s demonstration (in the pre-course survey) and instructional videos (in the post-course survey). The mean score of item 1 in the pre-course survey is 4.08 while that in post-course survey is 4.04. This shows that the students found class demonstration in the traditional teaching model more useful than instructional videos in the flipped classroom model. This finding disagrees with a previous finding that online videos are more useful (Love et al. 2014). One possible reason is that ways of presenting the materials may differ. The student teacher was still inexperienced. He may not have delivered the contents in an attractive way or explained the steps clearly in the instructional videos. This might have affected the results. Herreid and Schiller (2013) pointed out that one of the pitfalls of the flipped approach is that the “homework (readings, videos) must be carefully tailored for the students in order to prepare them for the in-class activities” (p.63). Table 5 also shows that not all students had done the pre-lesson video preparation for the four lessons. Only seven students had watched the instructional videos before each lesson and three never watched any at home.

**Table 4:** Scores of items assessing students’ perception of instructional videos and class tasks

	Number	Pre-course		Post-course		*t*	Sig.
		*M*	SD	*M*	SD		
Usefulness							
1. Teacher’s demonstration (traditional model)/instructional videos (flipped classroom)	25	4.08	.759	4.04	.735	.238	.814
2. Class task	25	3.40	.913	4.04	.735	−3.089	.005

**Table 5:** Completion of pre-lesson instructional videos

	Video completion (*N* = 28)				
	4 lessons	3 lessons	2 lessons	1 lesson	Never
No. of students	7	8	5	5	3

### Online quiz

In addition to the pre- and post-course surveys, the students were required to do an online quiz to see whether they were able to master the knowledge and skills after watching the instructional video for each lesson. However, as the student participation was on a voluntary basis, it was not possible to ensure that they completed the quiz for each lesson even when the student teacher had encouraged them to do so many times. Table 6 shows that the completion rate declined and dropped to 17.9% for lesson 4.

**Table 6:** Completion of the online quiz after each lesson

	Quiz completion (*N* = 28)			
	Lesson 1	Lesson 2	Lesson 3	Lesson 4
No. of students	16	8	6	5
% of students	57.1	28.6	21.4	17.9

The students’ performance is shown in Table 7. It is not surprising to see that they performed very well when tackling questions that required lower-order thinking skills such as *remembering* and *understanding*. However, some students had difficulty in answering questions that required higher-order thinking skills. Questions 5 and 6 of the lesson 4 quiz were designed to assess students’ ability to evaluate. These questions had more than one correct answer among the four options and the students were required to indicate all possible answers. Some students were only able to identify one of the two correct answers.

**Table 7:** Student performance in each online quiz

	Remembering	Understanding	Analysing	Evaluating	Overall
Lesson 1 (*N* = 16)					
Number of items	3	–	1	–	4
Correct %	96.7	–	85.0	–	93.8
Lesson 2 (*N* = 8)					
Number of items	4	–	–	–	4
Correct %	75.0	–	–	–	75.0
Lesson 3 (*N* = 6)					
Number of items	1	2	2	1	6
Correct %	50.0	91.7	8.3	66.7	52.8
Lesson 4 (*N* = 6)					
Number of items	1	3	–	2	6
Correct %	100.0	83.3	–	66.7	80.6
Overall					
Number of items	9	5	4	3	20
Correct %	82.2	86.6	50.4	66.7	73.8

### Students’ assignments

Before lesson 3, the students were required to submit assignment 1. The assignments were graded by the student teacher based on an assessment rubric. Students who demonstrated higher-order thinking ability according to the revised Bloom’s taxonomy were awarded a higher grade. This means that better-performing students could analyse the requirements of the 3D model task in detail and produce models in a creative way. On the other hand, if a student could only demonstrate low-level skills and fulfil the basic requirements, they got a fail grade. Table 8 shows the grade distribution for assignment 1. As the data for assignment 2 were to be collected after the field experience period, only the students’ performance in the first assignment can be presented here. The results show that 12 (42.8%) students got a good grade for their assignment. This indicates that the lesson objectives could be achieved to a certain extent through use of the flipped classroom approach.

**Table 8:** Student performance in assignment 1

	Grade					
	A	B	C	D	F	U
No of students	3	9	9	4	2	1
% of students	10.7	32.1	32.1	14.3	7.1	3.6

### Focus group interview

**Fig. 7:**
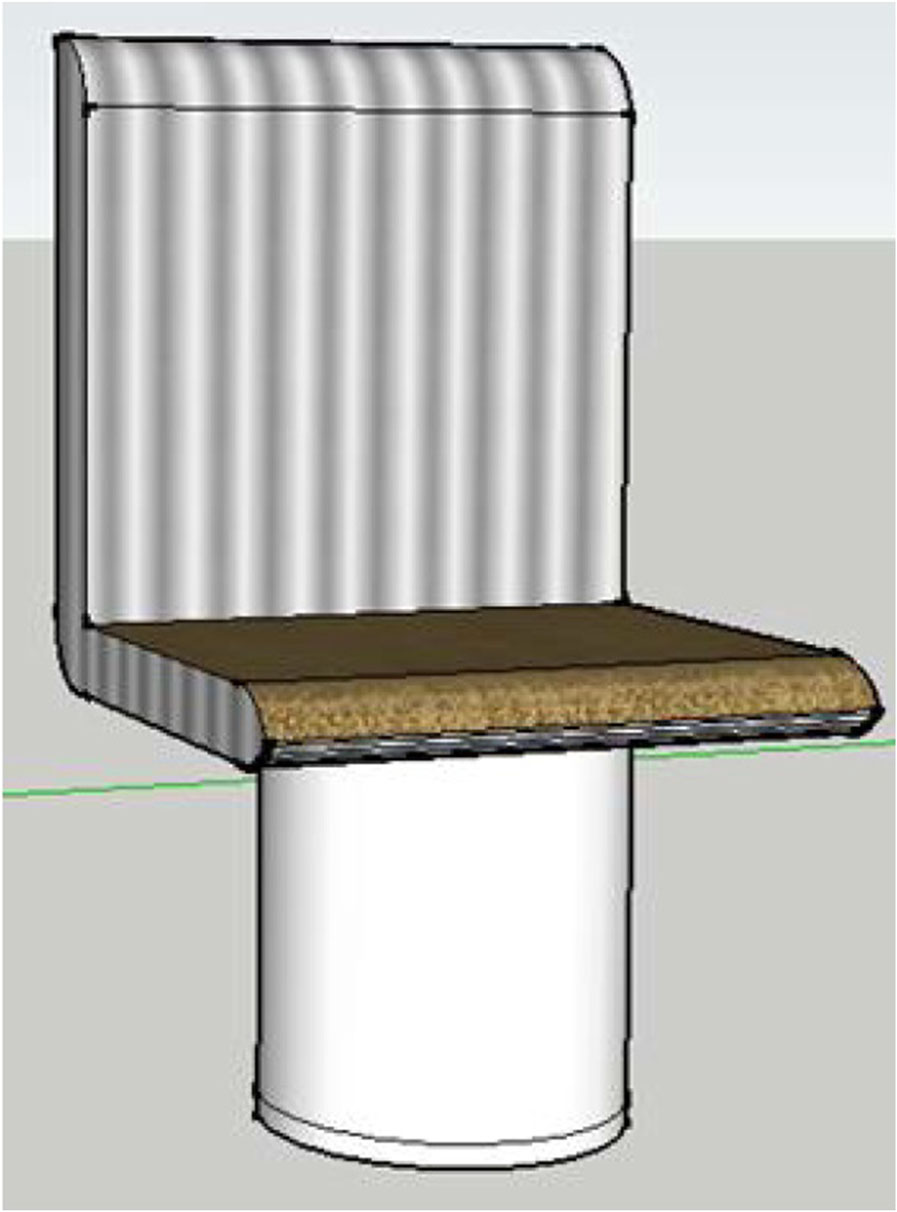
A model created by a student for assignment 1

In the interview, the students commented that the flipped classroom approach was more interesting as student-centred activities are more involving. One student thought that the approach should be adopted in core subjects and that the online resources were very useful when students missed classes. Another student who had demonstrated higher ability in *applying* and *analysing* skills in assignment 1 thought that the instructional videos helped him to solve complex problems. He also added that he was eager to explore different ways of using the tools provided by the software. Figure 7 shows the model drawn by this student for assignment 1. It demonstrates that he was able to use several tools and features provided by the 3D modelling software. His assignment was awarded a B grade as it fulfilled the task requirements and demonstrated a high level of creativity.

However, not all of the students considered the flipped classroom environment capable of nurturing higher-order thinking. One student reflected that:

My Form 1 Computer Literacy teacher told us that [learning to] use computer software does require us to explore its functionalities ourselves. However, in the flipped classroom model, we learned about the functions and operations of the 3D modelling software from the instructional videos. Therefore, we were no longer required to further investigate how to use that software. That’s why I don’t think that this approach will encourage students to learn deeper.

The student believed that learning to operate a new computer tool is an inquiry process, while the instructional videos provided too much information and were not better than a teacher’s demonstration. Some students even thought that the instructional videos reduced students’ motivation to explore the computer software. Surprisingly, this finding agrees with the Ho and Lam’s (2016) argument.

## Conclusions

The main objective of the study was to investigate the opportunities and challenges of implementing the flipped classroom model in secondary schools and to examine whether it is feasible to promote higher-order thinking using the flipped classroom approach. The findings led to answers to the study’s two main research questions, discussed below.

### RQ1: What are students’ perceptions of implementing the flipped classroom in public secondary schools in Hong Kong?

The survey findings show that most students felt that they could use the knowledge and skills taught by their teacher to start their projects. This was also reflected in their good performance in assignment 1. Moreover, as more student-centred learning activities could be conducted, some students commented that the new approach was less boring than the traditional approach. Most students also perceived that they could get more class support from their teacher during class time when using the flipped classroom approach. They agreed that more class time was available, which allowed them to complete the learning tasks in the class. In addition, they had more time to discuss with their teacher during the lesson. The flipped classroom can provide more class time for the teacher; it surely can enhance classroom interactions and help create a supportive learning environment.

However, it is still a great challenge to explore how to apply the flipped classroom approach in different subjects. For example, in this study, one student thought that students should have more freedom to explore how to use software tools. It also seemed that the students were not familiar with this teaching mode and some preferred a more traditional approach. These students preferred following teacher demonstrations in class time to watching instructional videos on the IT platform. Zainuddin and Halili (2016) point out that the problems of poor-quality video lectures and untrained instructors in the flipped classroom should be addressed by future researchers. This pitfall has also been mentioned by Herreid and Schiller (2013). Moreover, the findings in the previous section show that the number of students completing the pre-lesson video declined and that some never attempted to watch the instructional videos at home. Thus, when adopting the flipped classroom approach, students need more encouragement to adapt to the new mode of learning. It is too optimistic to assume that students will finish all assigned tasks without encouragement.

### RQ2: Is it feasible to promote higher-order thinking using the flipped classroom approach?

This study has shown that most students could analyse task requirements and designed models in creative ways. This can be observed in their performance in assignment 1 and their responses in the pre-course and post-course surveys. As the class activities in the study were not perfectly designed, they might have allowed some students to demonstrate only their lower-order thinking skills. The potential of the flipped classroom might not have been maximized and unleashed. It is an undeniable fact that the success of the flipped classroom approach heavily depends on lesson design. As the study was conducted by a student teacher during the field experience block practice period, the whole study period was limited to eight classes over 4 weeks. It was impossible to redesign all the learning activities and adopt an inquiry-based and student-centred approach. To have done so would have adversely affected the teaching process. Teachers should think carefully about how to develop students’ scientific inquiry ability in a flipped classroom. For example, students should be given more opportunities to explore their skills in using new technology and their understanding of the underlying concepts.

In any case, this empirical study provided the research team with concrete experience of applying the flipped classroom approach in real classroom settings. It seems that this approach gives teachers a new way of teaching that can be a superior alternative to traditional classroom-based learning. It would be worthwhile to explore how best to apply this new approach in different subjects at different levels.

## References

[CR1] Bergmann J, Sams A (2012). Flip your classroom: reach every student in every class every day [iTunes version].

[CR2] Bishop JL, Verleger MA (2013). The flipped classroom: a survey of the research. Paper presented at the 120th American Society for Engineering Education Annual Conference and Exposition, Atlanta, GA.

[CR3] Chua JSM, Lateef FA (2014). The flipped classroom: viewpoints in Asian universities. Education in Medicine Journal.

[CR4] Couch AC (2014). Comparison of teaching approaches and strategies: how do the use of traditional teaching and flipped classroom teaching techniques affect the attitudes and attainment of science students in an international school in Hong Kong?.

[CR5] Curriculum Development Council (CDC) (1999). Syllabuses for secondary schools: computer literacy (secondary 1-3).

[CR6] Education Bureau (2014). Consultation document on the fourth strategy on information technology in education: realising IT potential, unleashing learning power, a holistic approach.

[CR7] Hamdan, N., McKnight, P., McKnight, K., & Arfstrom, K. M. (2013). *A review of flipped learning*. Retrieved from http://flippedlearning.org/wp-content/uploads/2016/07/LitReview_FlippedLearning.pdf.

[CR8] Herreid CF, Schiller NA (2013). Case studies and the flipped classroom. J Coll Sci Teach.

[CR9] Ho, S. C., & Lam, Y. P. (2016, December 19). 如何解讀香港PISA 2015科學科表現? Mingpao. Retrieved from http://mingpao.com [In Chinese]

[CR10] Johnson L, Adams Becker S, Estrada V, Freeman A (2014). NMC horizon report: 2014 higher education edition.

[CR11] Khairuddin, N. N., & Hashim, K. (2008). Application of Bloom's taxonomy in software engineering assessments. In Misra, S. C., Revetria, R., Sztandera, L. M., Iliescu, M., Zaharim, A., & Parsiani, H. Recent Advances in Applied Computer Science. Paper presented at the 8th WSEAS International Conference on Applied Computer Science (ACS'08), Venice (pp. 66-69). (n.p.): WSEAS Press.

[CR12] Krathwohl DR (2002). A revision of Bloom’s taxonomy: an overview. Theory Pract.

[CR13] Lai Y, Wong T (2005). Develop pupils’ higher-order thinking abilities via creative teaching approach. Hong Kong Journal of Early Childhood.

[CR14] Lankford L (2013). Isn’t the flipped classroom just blended learning? [Blog post].

[CR15] Leung LL (2013). An inquiry of teachers’ perception on the relationship between higher-order thinking nurturing and liberal studies public assessment in Hong Kong. Teachers’ Centre Journal.

[CR16] Lo K (2016). Hong Kong slips to new low in international ranking for student performance in science, South China morning post.

[CR17] Love B, Hodge A, Grandgenett N, Swift AW (2014). Student learning and perceptions in a flipped linear algebra course. Int J Math Educ Sci Technol.

[CR18] Nederveld A, Berge ZL (2015). Flipped learning in the workplace. J Work Learn.

[CR19] Schreiber J, Asner-Self K (2010). Experimental and nonexperimental research design. Educational research: The interrelationship of questions, sampling, design, and analysis.

[CR20] See S, Conry JM (2014). Flip my class! A faculty development demonstration of a flipped-classroom. Currents in Pharmacy Teaching and Learning.

[CR21] Smith I (2007). How to ask better questions.

[CR22] Yeung SY (2012). Conceptualizing higher-order thinking for reforming school curriculum and teaching. Hong Kong Teachers’ Centre Journal.

[CR23] Zainuddin, Z., & Halili, S. (2016). Flipped classroom research and trends from different fields of study. The International Review of Research in Open and Distributed Learning, 17(3). doi:10.19173/irrodl.v17i3.2274

